# Nutrient supplementation strategy improves cell concentration and longevity, monoclonal antibody production and lactate metabolism of Chinese hamster ovary cells

**DOI:** 10.1080/21655979.2020.1744266

**Published:** 2020-03-30

**Authors:** Saumel Pérez-Rodriguez, María de Jesús Ramírez-Lira, Mauricio A. Trujillo-Roldán, Norma A. Valdez-Cruz

**Affiliations:** Programa de Investigación de Producción de Biomoléculas, Departamento de Biología Molecular y Biotecnología, Instituto de Investigaciones Biomédicas, Universidad Nacional Autónoma de México, Coyoacán, Ciudad De México, México

**Keywords:** PowerFeed A, Chinese hamster ovary cells, monoclonal antibody production, cellular growth, metabolism

## Abstract

A careful selection of culture mediums and feeds has become necessary to maximize yields of recombinant proteins during bioprocesses of mammalian cells. Supplements contain a variety of concentrate nutrients, and their beneficial effects vary according to recombinant cell lines. In this study, the effects of PowerFeed A on growth kinetics, productivity and cellular metabolism were evaluated for two Chinese hamster ovary cell lines producing a monoclonal antibody in a batch culture. Supplemented cultures increased integral viable cell density of CRL-12444 and CRL-12445 cells by 2.4 and 1.6 times through extension of culture time at which viability was above 90% in 72 and 36 h, respectively, and increment of maximal cell concentration in 3.25 × 10^6^ cells/ml (69%) for CRL-12445 cells. Product titer augmented 1.9 and 2.5 times for CRL-12444 and CRL-12445 cells, respectively, without changes in growth rate and specific productivity. Feed supplementation also stimulated full consumption of glucose and free glutamine and reduced 10 times lactate accumulation, while ammonium, sodium and potassium remained at similar concentrations at the end of the culture. About 44% of calcium, mainly provided by feed, was consumed by both cell lines. Maximization of cellular growth, viability and protein titer through feeding encourages extending its use to other cell lines and exploring novel combinations with other basal mediums or feeds. A thorough investigation of its impact on protein quality and the molecular mechanisms behind these effects will allow designing effective feeds and strategies to rationally optimize protein production in the biomanufacturing industry.

## Graphical Abstract

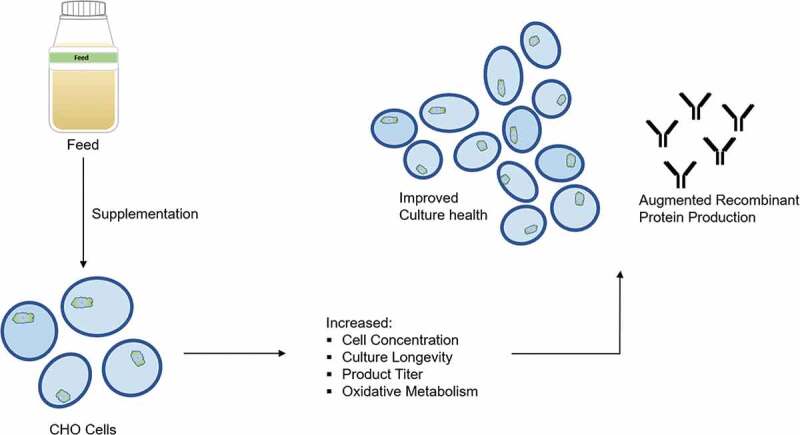

## Introduction

Chinese hamster ovary (CHO) cells have been widely employed for expression of recombinant proteins (RPs); indeed, most of the approved human therapeutic antibodies (84%) have been produced in this cellular platform [1]. Therefore, given the biotechnological value of these cells, the development of new culture media, feeds and culture methods and the improvement of those already used for their culture are a continuous process [2]. Progress in the culture media for biopharmaceutical production of CHO cells performed for about 40 years has resulted in the current use of a fully chemically defined growth medium, with non-animal-derived substances, that minimize changes batch to batch, allowing the reproducibility and enhancing the productivity [3]. The composition of the serum-free culture medium that is used for CHO cells as a reference has been previously reported [4,5]. However, the composition of the many culture media adapted to different cell lines, following different gene expression strategies, to obtain different RPs with particular characteristics, and used in the manufacture of biopharmaceuticals, is not normally disclosed for commercial reasons [6]. The development of the culture medium and feeds and their uses in bioprocesses are of the utmost importance for the cell culture experiments, as well as for biopharmaceutical work. In fact, the optimization of the composition of culture media and feeds has a direct and positive impact on the duration of the bioprocesses [7–18], productivity [7–22] and purification and quality of proteins [9–11,13,15,17,22], factors that ultimately impact on lower RP costs. Feeds have been designed to replenish depleted nutrients in basal medium, alleviating different cellular stresses and supporting the cell growth and RP production; they can be classified into those of general use [7,8,10,12,15–17,19,20] or those specifically developed for certain clones [3,18,21,22]. Although these supplements are usually added to culture media during the course of the bioprocess (fed-batch culture) [3,10–12,14–16,18–22], they can be used at the beginning of the culture (batch culture) as well [3,7,8,13,17]. Feeds can be classified as chemically defined nutrient cocktails (CDF) or concentrated hydrolyzates; in the first case, their exact composition is generally not available excluding some exceptions [3,18,21,22], and in the second one, they remain as black boxes for their composition. They have demonstrated a positive impact on cell concentration, viability, product titer, specific and volumetric productivity and metabolic behavior during the production of different RPs such as monoclonal antibodies (MAbs) [8–13,15-23], blood clotting factors [14] and fusion proteins [3], or in non-transformed cells [7,23], without significant changes in protein quality [13,17,20,22]. Although many efforts have focused on the development of supplements that can be used in several cell lines, the nutritional requirements are specific to each of them [9,12,13,15,21,22,24,25], so the benefits of feeds should be evaluated on a case-by-case basis. Designing feeds toward CDF has been a strong tendency adopted in the last years for a tight control over process optimization [12].

Despite being usually proprietary formulations, CDFs are intensively employed for the production of RP, at both research and production facilities, highlighting the utmost importance of characterizing their effects on cell biology, RP expression and protein quality and the mechanisms by which these effects are achieved. One of these CDFs is PowerFeed A, a non-animal origin, serum- and protein-free supplement designed to support growth and protein expression of suspension CHO cells.

Various proved advantages of feeds on culture performance and RP production in CHO cells have been reported, although the improvements are related to cell lines, products and culture strategies [3,13,15]. Then, the goal of the present research is to evaluate the impact of this supplement on cell growth, RP production and metabolism of two CHO cell lines expressing the same MAb at different specific productivity. To our best knowledge, this is the first report that evaluates the effects of this supplement during the culture of suspension recombinant CHO cells, pointing out its utility and positive impact on the biotechnological processes [11,17,18].

## Materials and methods

### Cell lines and culture conditions

Cell lines CHO DP-12 clones #1933 (CRL-12444) and 1934 (CRL-12445) ATCC® (Manassas, VA, the USA) [26] were used in this work. Both cell lines were adapted to grow in suspension, and both produce humanized anti-IL-8 MAbs in different proportions. Both cell lines were cultured in CDM4CHO medium (GE Healthcare, Chicago, IL, the USA) supplemented with 6 mM stable glutamine (Biowest LLC, Nuaillé, France), 0.002 mg/ml Humulin N (Eli Lilly, Indianapolis, IN, the USA) and 200 nM methotrexate (Pfizer, New York, NY, the USA), at 37ºC in a 5% CO_2_ atmosphere in a humidified incubator (Model NU-5500, Nuaire, Plymouth, MN, the USA). The culture medium was supplemented with 20% (v/v) PowerFeed A (BE02-044Q, Sartorius AG, Göttingen, Germany).

The inoculum was expanded in 75 cm^2^ T-flasks at an orbital agitation of 60 rpm (Cat. 7644–10115 Bellco Glass Inc., Vineland, NJ., the USA), and cells were seeded at 0.30 × 10^6^ cells/ml in duplicate in 25 cm^2^ T-flasks with a filled volume of 8 ml. Cell concentration and viability were recorded by cell counting in a Neubauer chamber using the trypan blue dye exclusion method (Model AE2000, Motic, Hong Kong, China) [27].

### Quantification of metabolites, ions and pH

Glucose, lactate, glutamine, glutamate, ammonium, sodium, potassium and calcium concentrations in culture supernatants were measured at the end of cultures by using BioProfile® FLEX2™ Automated Cell Culture Analyzer (Nova Biomedical, Waltham, MA, the USA).

### Quantification of overall specific productivity

For calculation of overall specific productivity (Qp), IgG concentration in supernatants was measured at the end of cultures by using Human IgG enzyme-linked immunosorbent assay Quantitation Set (E80-104, Bethyl Laboratories, Inc., Montgomery, the USA), according to manufacturer's protocol. SigmaFast o-phenylenediamine dihydrochloride substrate (Merck KGaA, Darmstadt, Germany) was prepared according to the manufacturer’s recommendations and incubated at room temperature for 15 min. The enzymatic reaction was stopped by the addition of 50 µl of HCl 10% (v/v) per well and absorbance recorded at 490 nm (Stat Fax 4200, Grupo Mexlab, Zapopan, Jalisco, Mexico).

Overall Qp was calculated according to the formula Qp = ΔP/IVCD, where ΔP is the antibody concentration at the end of culture [11]. Integral viable cell concentration (IVCD), understood as the concentration of viable cells that were working during the time the cell culture lasted, was calculated as area under curve by trapezium rule using GraphPad Prism Software v5.01 (GraphPad Software Inc., San Diego, CA, the USA).

### Image processing

GraphPad Prism v5.01 was employed for the construction of growth kinetics and metabolite graphics.

### Statistical analysis

All evaluated parameters were compared between the two tested conditions (nonfeed supplemented versus supplemented) for each cell line by using the non-parametric Mann–Whitney test in GraphPad Prism v5.01.

## Results

**Figure 1.**
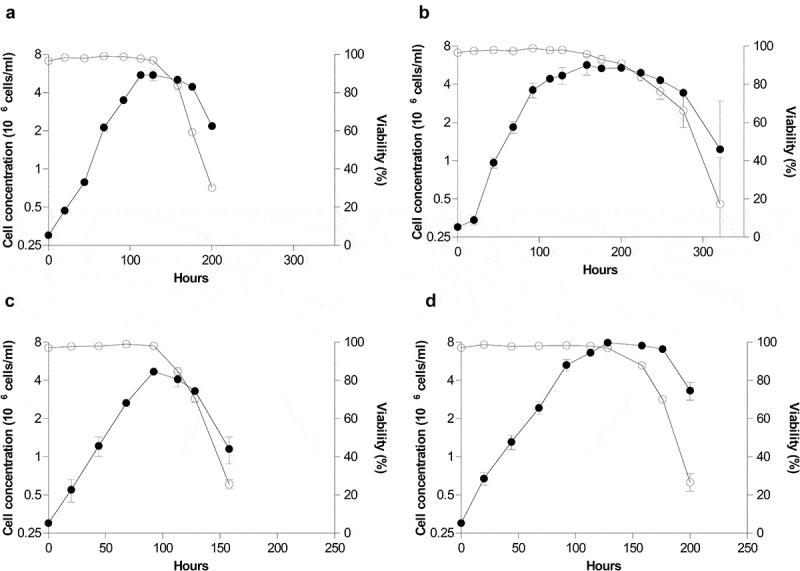
Effects of PowerFeed A on growth kinetics of Chinese hamster ovary cells producing a monoclonal antibody. CRL-12444 (a, b) and CRL-12445 (c, d) cells were seeded by duplicate at 0.3 × 10^6^ cells/ml in 25 cm^2^ T-Flasks, with (b, d) or without (a, c) the initial addition of 20% (v/v) of feed. Viable cell concentration (closed circles) and viability (open circles) of cultures were determined over time by trypan blue dye exclusion method in a Neubauer chamber. Error bars represent the standard deviation of two biological replicates.

The beneficial effects of media supplementation on humanized anti-IL-8 MAbs production by CHO cells were tested in the cell lines CRL-12444 and CRL-12445, previously adapted to grow in suspension, during batch cultures in 25 cm^2^ T-flasks. The culture of lower producer CRL-12444 cells (Figure 1(a,b)), under control and supplemented conditions, showed a maximal cell concentration of 5.74 ± 0.23 vs 5.98 ± 0.53 × 10^6^ cells/ml and specific growth rate of 0.029 ± 0.000 vs 0.032 ± 0.001 h^−1^, respectively. Maximal cell concentration and specific growth rate remained unchanged despite feed addition, whereas culture time at which viability was above 90% was substantially extended from 128 to 200 h (Figure 1(b)). On the other hand, in case of higher producer CRL-12445 cells (Figure 1(c,d)), although the specific growth rate was unaffected as well (0.030 ± 0.004 vs 0.028 ± 0.000 h^−1^), maximal cell concentration increased from 4.70 ± 0.15 to 7.95 ± 0.35 × 10^6^ cells/ml, and culture time at which viability was above 90% increased from 92 to 128 h due to media supplementation. As a consequence of the elevated cell concentration and extended viability, the IVCD increased 2.4 (26.06 ± 2.14 vs 41.73 ± 4.75 × 10^6^ cell/ml day) and 1.6 (15.79 ± 1.90 vs 37.27 ± 2.95 × 10^6^ cell/ml day) times for CRL-12445 and CRL-12444 cells, respectively. Furthermore, product titer, quantified in culture supernatants, was also improved in 1.9 (3.15 ± 1.21 vs 6.52 ± 0.75 ng/ml) and 2.5 (244.40 ± 13.80 vs 604.50 ± 79.80 ng/ml) times for CRL-12444 and CRL-12445 cells, respectively, cultured under supplemented conditions. This together with higher IVCD values resulted in no net changes in overall Qp for either cell line (0.13 ± 0.02 vs 0.16 ± 0.00 and 15.54 ± 0.99 vs 16.18 ± 0.84 × 10^−3^ pg/cell day for CRL-12444 and CRL-12445 cells, respectively).

**Figure 2.**
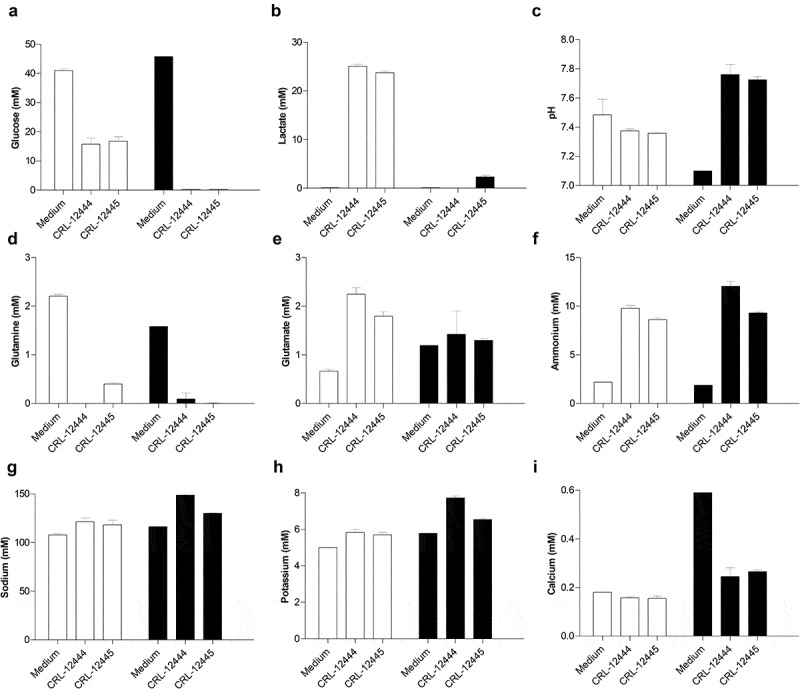
Effects of PowerFeed A on metabolism of Chinese hamster ovary cells producing a monoclonal antibody. Concentration of key metabolites (glucose [A], lactate [B], glutamine [D], glutamate [E], ammonium [F]), pH (c) and ions (sodium [G], potassium [H], calcium [I]) in non-spent medium and supernatants of CRL-12444 and CRL-12445 cells were measured at the end of batch cultures in 25 cm^2^ T-Flasks, with (black columns) or without (white columns) the initial addition of 20% (v/v) of feed. Metabolites, pH and ions were quantified by using BioProfile® FLEX2™ Automated Cell Culture Analyzer. Error bars represent the standard deviation of two biological replicates.

Besides cell growth and MAb production, cellular metabolism was also analyzed by measuring key metabolites in supernatants at the end of the cultures (Figure 2). Under no supplementation conditions, both cell lines consumed around 60% of glucose (Figure 2(a)) and produced 24.5 mM lactate on average (Figure 2(b)), acidifying culture medium in 0.12 pH units (Figure 2(c)). On the contrary, when the culture medium was supplemented, available glucose was exhausted from supernatants from both cell lines (Figure 2(a)), negligible (2.33 ± 0.31 mM) or no amounts of lactate were accumulated (Figure 2(b)) and pH raised on 0.64 units on average (Figure 2(c)). In the case of glutamine, it was provided as a free amino acid (≈2 mM) and as an alanyl-glutamine dipeptide (6 mM) where only the free amino acid could be measured. In all conditions, free glutamine was almost completely consumed from extracellular medium (Figure 2(d)), generating glutamate (Figure 2(e)) and ammonium as a toxic byproduct (Figure 2(f)) [28–34]. In the case of feed supplementation, cumulative glutamate levels decreased by 37% and 27% in CRL-12444 and CRL-12445 cells, respectively, compared with glutamate accumulation in control cultures (Figure 2(e)). In the same sense, CRL-12444 accumulated 23% more ammonium at the end of the culture under supplemented strategy (Figure 2(f)). Given the high importance of salts like sodium, potassium and calcium for several cellular processes such as transmembrane potential, nutrition, buffering, osmolality and signal transduction [2], their concentrations were measured. No changes in sodium (Figure 2(g)) and potassium (Figure 2(h)) levels were detected for any condition. The quantification of sodium and potassium indicates only a small increase in the concentration of both salts after medium supplementation. Whereas around three times of calcium concentration (0.59 mM) was quantified in supplemented media compared with media control, 0.33 mM of calcium was consumed by both cell lines exclusively under feed supplementation (Figure 2(i)).

## Discussion

The understanding and evaluation of which culture medium can be beneficial for each cell line in growth, viability and productivity define its possible usefulness in the industry, although this evaluation is still an empirical search. During cell development, it is normal to work with chemically defined media without a knowledge of the different formulas of the media which normally are patented and whose description is not public. Currently, several studies have been conducted to deeply understand the molecular events under different environmental conditions that are totally influenced by the culture media and their effect on metabolism [3,8,35]. Several supplements for production of RP in CHO cells have been developed and characterized, whether chemically defined or not [3,8,10,12,14-16,18-22]. On the contrary, the feed used in this work has not been characterized so far in the context of RP production by mammalian cells.

The initial supplementation of this feed to culture medium greatly extended cell longevity for cell lines CRL-12444 and CRL-12445, and in case of the higher producer one, maximal cell concentration as well, without perturbing specific growth rate and overall Qp (Figure 1). As a consequence of a larger number of viable cells and culture viability, IVCD of both cell lines significantly increased in comparison with non-supplemented control, which translated into higher product titers without altering the quantity of MAb secreted by each cell at each time (Qp). In agreement with these results, a significant increment in maximal cell concentration, culture viability and MAb titer has been continuously reported for CDFs during batch and fed-batch cultures of CHO cells [3,8–11,13,15,16,18,21–23]. In most cases, both viability and maximal cell concentration contributed to higher product titers; however, occasionally only one of them does [8,21], as in the case of our lower cell line producer (CRL-12444). Occasionally, feed supplementation can augment the three parameters that at the end support product titer, that is cell concentration, viability (longevity) and Qp [11]. On the contrary, the specific growth rate has been demonstrated to be poorly affected by feeds [7,15], whereas Qp increase depends largely on cell line and bioprocess [8,13,15,17]. It has been reported that certain combinations of feeds have increased IVCD and MAb titers of CHOK1 IgG1-producing cells by 85% and 170% in batch cultures, respectively, and maintained higher IVCD, MAb titers and viability during the exponential growth phase in small-scale perfusion cultures [13]. In both scenarios, cultures increased product titer by accumulating biomass and extending process duration, as for supplemented higher producer CRL-12445 cells.

Besides growth parameters and productivity, key metabolites from glycolysis and glutaminolysis, two essential routes for CHO cells [28], and important salts were measured as well. Metabolic behavior of both cell lines was improved by feed in terms of glucose consumption and lactate accumulation (Figure 2), indicating a shuttling from a glycolytic to respiratory metabolism. Although it has been reported previously that lactate consumption is directly linked to a phenotype of higher Qp and oxidative activity [36,37], Qp does not change in any experimental condition tested here.

In line with other reports [25,38,39], the low accumulation of lactate at the end of the cultures is directly influenced by the medium composition and probably occurs as a result of a lactate consumption to maintain mitochondrial oxidative capacity, viability and energy requirements [12,38]. Since all metabolites were measured at the end of cultures, it is not possible to distinguish clearly if the lactate was not produced at all or it was produced and later consumed in supplemented cultures. Previously, it has been reported that certain supplements could induce a lactate consumption phenotype [12,15,38]. Feed effects on lactate levels could be caused by glucose depletion due to higher IVCD values, leading to the known switch in lactate metabolism in basal media [13,38] or supplemented cultures [9,10]. Lactate accumulation has a negative influence on growth, specific productivity, product titer, oxygen consumption, viability and survival of baby hamster kidney **cells (**BHK) [29], murine hybridoma [40] and CHO cells [32,41], although these effects and its inhibitory concentration are cell line specific. These findings highlight the importance of using supplements that prevent lactate accumulation during bioprocesses, like those that also maintain low lactate levels facilitating a more efficient consumption of glucose and glutamine and contributing to higher maximal cell concentration, MAb titer and product quality [32,41].

As glutamine was only measured as a free amino acid and was initially provided as a dipeptide, it was not possible to determine its total levels. However, because higher glutamate concentrations provided by feed remained unchanged at the end of cultures (Figure 2(e)), it appears that this additive stimulates complete oxidation of free glutamine to α-ketoglutarate in order to replenish key intermediates from tricarboxylic acid cycle. Since glutamine has demonstrated to be necessary to sustain cell growth and product titer for most cells and specific productivity depending on cell line [11,34,42], it is expected that supplemented cultures consumed this amino acid at higher rates.

Even though sodium and potassium ions are very important in cell nutrition and transmembrane potential, feed supplementation did not enrich the concentration of these ions. Among the measured ions, only calcium was provided by feed and readily consumed during cultures. It has been demonstrated that absence or the overload of intracellular calcium may trigger apoptotic cell death [43]. Then, calcium added by the feed could participate in prolonging culture time and in the maintenance of the productivity of RP in the cells and contribute to the more robust producer phenotype [43–46].

According to growth kinetics, Qp and concentrations of metabolites, this supplement alters the metabolism and probably cell cycle but not gross mechanisms that control translation and secretion of proteins. Given that other supplements used during CHO cell cultures have not altered product quality in terms of aggregation, charge variants, glycoforms and molecular weight forms [13,17,20,22], it is expected that this feed has not changed MAb quality.

Systems biology approaches have been recently applied to the design and optimize culture media and feeds to comprehensively understand the cellular mechanisms deregulated after adaptation to new media or supplementation [47]. Transcriptomics [17], proteomics [3,18] and metabolomics [3,21] studies have been carried out to identify key targets for tailored feeding, genetic engineering and design of a new generation of supplements from the previous ones. These same omics could be applied in the future to explain observed differences in carbon and nitrogen metabolism, calcium-dependent processes and cellular growth and to discard perturbations on translation machinery and classical secretion pathway, observed with the feed here studied.

Given the positive effects of the supplement used in this work on CHO cellular growth and MAb production, it is advisable to expand the current knowledge to other proteins, cell lines and culture strategies, evaluate the key metabolites kinetically and explore the cellular mechanisms responsible for the observed phenotypes.

## Conclusions

The characterization of new supplements plays a crucial role in the sustained increment of RP production by CHO cells. Effects of PowerFeed A on growth kinetics, Qp and cellular metabolism were evaluated for the first time for two CHO cell lines producing an MAb in a batch process. Supplemented cultures increased maximal cell concentration by 69% and cell longevity beyond 36 h while maintaining viability above 90%, resulting in approximately twice MAb titers. The specific growth rate and Qp were not affected. Although feed addition led to glucose depletion, low levels of lactate were accumulated at the end of the culture. Additionally, feed stimulated glutamine and calcium consumption. The data presented here may be used to augment the production of MAbs and other therapeutic proteins during heterologous expression in CHO cells and probably may be extended to other mammalian cells. Together, it emphasizes that the design of culture media and supplements, as well as their evaluation carried out as an empirical search, allowed us to find the combination that can improve the growth of CHO cell lines and accumulation of an MAb. The supplementation of the production culture medium could favor the anaplerotic TCA pathways and overcome lipid limitations, resulting in favorable effects observed during MAb production. The data found encourage the design of new media and supplements with the intention of improving bioprocesses, and omics approaches will shed light on the cellular effects exerted by this supplementation on recombinant CHO cells.

## Research highlights

- Supplementation increased cell concentration and lifespan depending on the cell line
- Higher cell density and longevity contributed to increase product titer
- Feed addition reduced lactate accumulation in supernatants
- Supplementation stimulated calcium consumption
- Growth rate and specific productivity were unchanged despite supplementation
